# The effect of two novel cholesterol-lowering agents, disodium ascorbyl phytostanol phosphate (DAPP) and nanostructured aluminosilicate (NSAS) on the expression and activity of P-glycoprotein within Caco-2 cells

**DOI:** 10.1186/1476-511X-13-153

**Published:** 2014-10-01

**Authors:** Kristina Sachs-Barrable, Jerald W Darlington, Kishor M Wasan

**Affiliations:** Division of Pharmaceutics and Biopharmaceutics, Faculty of Pharmaceutical Sciences, The University of British Columbia, 2405 Wesbrook Mall, Vancouver, V6T 1Z3 British Columbia Canada; AMCOL International Corporation, Chicago, USA; Drug Discovery and Development Research Group, College of Pharmacy and Nutrition, University of Saskatchewan, Saskatoon, Saskatchewan Canada

## Abstract

**Background:**

Many drugs are substrates for P-glycoprotein (P-gp) and interactions involving P-gp may be relevant to clinical practice. Co-administration with P-gp inhibitors or inducers changes the absorption profile as well as the risk for drug toxicity, therefore it is important to evaluate possible P-gp alterations. The purpose of this study was to investigate the effect of two novel cholesterol-lowering agents, disodium ascorbyl phytostanol phosphate (DAPP) and nanostructured aluminium silicate (NSAS), a protonated montmorillonite clay, on *mdr-1* gene expression and its protein, P-glycoprotein (P-gp) within Caco-2 cells.

**Methods:**

The effects of DAPP and NSAS on the regulation of *mdr-1* gene, P-gp protein expression and activity within Caco-2 cells, were determined using cell viability and cytotoxicity tests, RT-PCR, Western Blot analysis and bi-directional transport studies.

**Results:**

We observed a significant down-regulation of *mdr-1* mRNA (e.g. 38.5 ± 17% decrease vs. control at 5 μM DAPP and 61.2 ± 25% versus control at 10 μM DAPP; n = 6, P* < 0.05) within Caco-2 cells. Western Blot analysis of P-gp expression showed that changes in *mdr-1* gene expression lead to correlating changes in P-gp protein expression. This down-regulation of P-glycoprotein also resulted in decreased activity of P-glycoprotein compared to untreated control. In contrast, when Caco-2 cells were treated with NSAS, no changes in *mdr-1* gene expression, P-gp protein expression nor P-gp activity were observed.

**Conclusions:**

DAPP but not NSAS decreases P-gp mediated drug efflux through decreased *mdr-1* gene expression and consequently decreased P-gp protein expression. These findings have to be taken into consideration when DAPP is concurrently given with other drugs that are substrates for P-gp since drug-drug interactions harbour a safety issue and alter bioavailability profiles.

NSAS does not have any P-gp altering properties and therefore might not affect drug-drug interactions. We conclude from this study that NSAS might make a safer drug candidate compared to DAPP for lowering LDL-cholesterol.

## Introduction

It has been estimated that by 2030 the number of deaths due to Cardiovascular disease (CVD) will climb to 23.3 million and CVD will remain the single leading cause of death [1]. The World Health Organization recommends increased government investment in prevention and early detection through national programs that are aimed to prevent and control non-communicable diseases like CVD. The application of new research findings towards new and better treatments, personalized medical care and interventions as well as the implementation of updated and improved guidelines are necessary for better outcomes for patients.

Substantial scientific evidence highlights elevated cholesterol levels as a risk factor for coronary artery disease. Despite much clinical success, statins are not well tolerated by all patients. Sufficient LDL-cholesterol lowering cannot be achieved by statin monotherapy in every patient [2–4] and reduction of cardiovascular events can only be reduced by 33% in the most responsive patients [5]. In about 2-10% of patients on statins the side effects are very severe and therefore the statin therapy has to be discontinued and replaced with alternative therapies [6]. An alternative approach to decreasing LDL-cholesterol is inhibiting the absorption of dietary cholesterol using plant sterols and plant stanols (phytosterols and phytostanols), either as as dietary supplements or as additives in food products including margarine, cereals, juices and yogurt [7]. The ability of plant sterols to reduce serum cholesterol levels has been known since the 1920s and was confirmed by studies in animals and humans in the 1950s [8–10].

Despite considerable research, the mechanism by which they reduce cholesterol is a topic of debate. Phytosterols are poorly absorbed in the intestines (between 0.4% and 3.5%), and as with cholesterol, they are poorly water soluble [11].

One explanation is that phytosterols compete with cholesterol for being incorporated into mixed micelles which are made of dietary fat, bile acids and sterols. Cholesterol not incorporated into the micelle phase is unable to cross the unstirred water layer that lines the intestinal wall and cannot be taken up by the enterocytes for subsequent packaging into chylomicrons. Other studies are suggestive of alternate mechanisms contributing to their LDL-cholesterol lowering characteristics. Kaneko *et al.* have proposed that phytosterols might function as agonists for the liver X receptor (LXR). LXR is a nuclear receptor responsible for upregulating cholesterol efflux pathways throughout the body [12]. Additionally, suppression of *de novo* cholesterol synthesis has been shown in a rat model [13].

**Figure 1:**
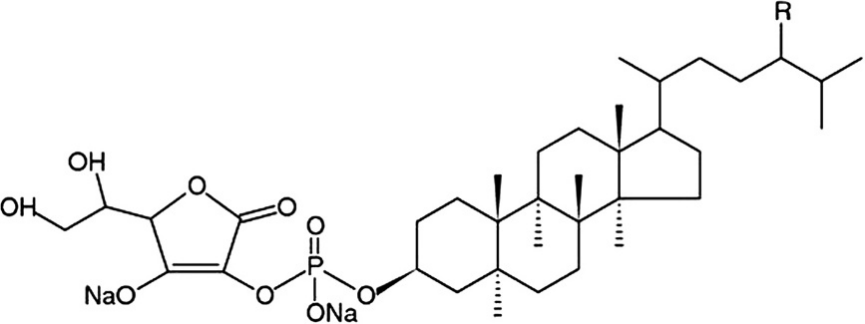
**Chemical structure of DAPP.** The two major components of DAPP are disodium ascorbyl campestanol phosphate and disodium ascorbyl sitostanol phosphate, each covalently linked to ascorbic acid by a phosphodiester bond [14, 15].

Phytosterols can be modified to increase their micellar incorporation capacity by esterification with fatty acids and many different modified phytosterols have been tested for activity. One such well characterized phytosterol is disodium ascorbyl phytostanol phosphate (DAPP), also known as FM-VP4 (Figure 1). It is a water-soluble derivative of sitostanol and campestanol linked by esterification with an ascorbyl-phosphate group [14, 15]. In animal studies with rats, gerbils and mice FM-VP4 effectively reduces dietary cholesterol absorption [16, 17]. This cholesterol lowering effect also leads to a reduction of atherosclerotic lesion formation in apo E knockout mice, a model that represents atherosclerosis [18]. FM-VP4 has also shown a decrease in body mass without any toxic effects [19]. This dose dependent reduction of mass in mice treated with FM-VP4 was not due to an increase in resting metabolic rate or decreased food or water intake, but through decreased absorption or increased excretion of lipids.

Potent cholesterol-lowering characteristics without any significant toxic effects was also shown in preclinical and clinical studies [15, 16]. A clinical study in 30 men demonstrated that up to 800 mg/day of DAPP is safe and well tolerated for at least 4 weeks. LDL-cholesterol was significantly reduced by 6.6% (p = 0.02) in the 400 mg per day group [20].

Another compound of interest is nanostructured aluminosilicate (NSAS). NSAS is a protonated montmorillonite (bentonite) clay. Bentonites are naturally occurring compounds and recent studies have demonstrated cholesterol lowering effects and define them as a new class of cholesterol absorption inhibitors. NSAS can reduce the absorption of cholesterol by 39%, similar to an identical dose of stigmasterol, by competing with cholesterol for absorption in the intestine after ingestion [21]. The first reported medical use of bentonite clay goes back to ancient Mesopotamia where it was applied externally as a mud bath and was touted for anti-inflammatory and antiseptic characteristics.

**Figure 2:**
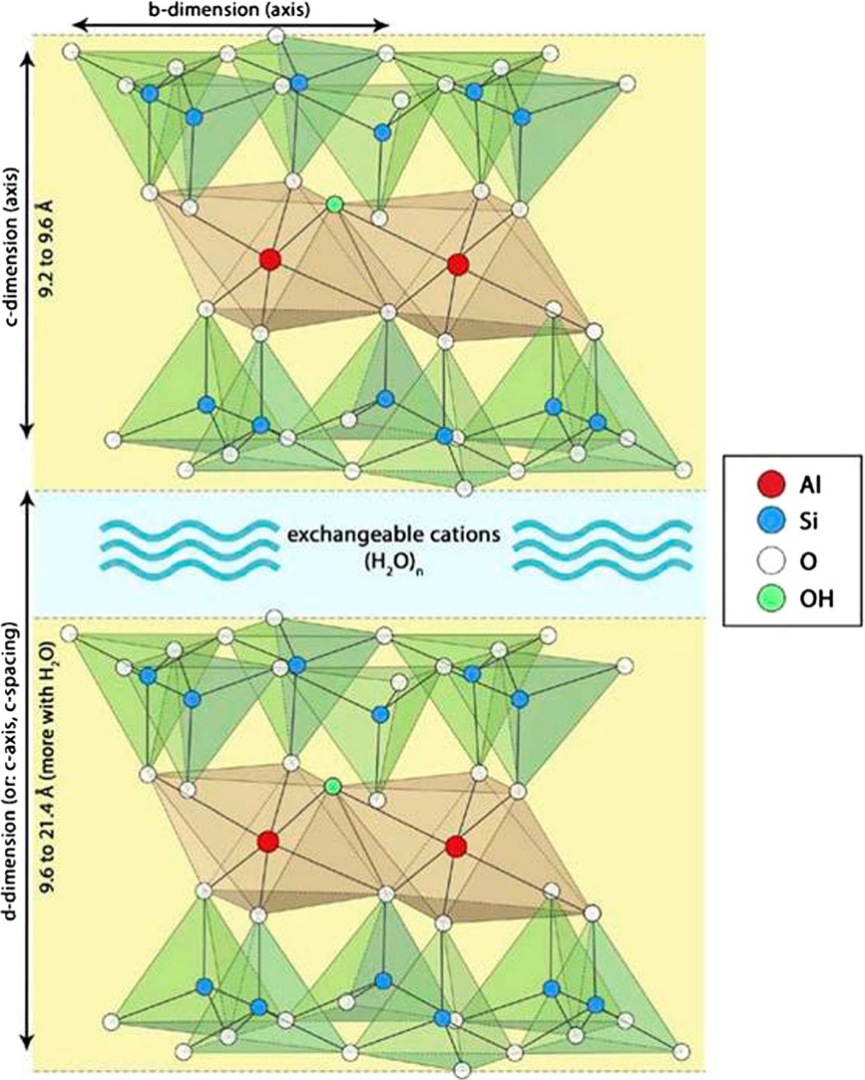
**Structure of montmorillonite clay.** Montmorillonite has a layer-lattice structure consisting of two sheets of tetrahedral siliconcrystals enclosing a sheet of octahedral aluminium crystals. Water and surface cations enter between adjacent silicon sheets causing the material to expand.

NSAS is composed of layers of aluminium octahedral sheets sandwiched between two silicon-oxygen tetrahedral layers (Figure 2). NSAS has a high surface area (200-800 m^2^/g) and shows high water and organic material absorption characteristics either by adsorption onto its external surface or into its interlaminar space. It is negatively charged in contrast to positively charged bile acids and surface protons are incorporated to counterbalance platelet surface negative charge [22]. Additionally, NSAS appears relatively safe for oral administration and is minimally absorbed.

Oral administration of NSAS significantly inhibits cholesterol absorption in mice. Apo E knockout mice fed a diet high in cholesterol and fat were treated for 12 weeks with NSAS (1.4% w/w) or stigmastanol (2% w/w) and showed significant reductions in plasma cholesterol concentrations relative to control animals in both groups. No changes in food and water intake or body weight were observed. Atherosclerotic lesion formation at the aortic root was also reduced [23]. *In vitro* lipolysis studies adapted to stimulate intraluminal processing of triglycerides and cholesterol in the presence of NSAS showed an inhibition of cholesterol absorption either by direct binding to cholesterol or by other mechanisms like incorporation into micelles. Protonated NSAS adsorbs and sequesters cholesterol from the aqueous phase in the digestive tract resulting in precipitation and excretion with feces [24]. Potent inhibition of cholesterol absorption and minimal systemic exposure make NSAS a considerable candidate for treatment of hypercholesterolemia, either as adjunctive therapy with statin or as monotherapy in patients unable to tolerate statins.

Most cardiovascular patients require multiple medications in addition to lipid lowering therapy, creating the possibility for drug interactions. Many of these drugs are substrates for P-glycoprotein (P-gp), which mitigates cellular exposure to various hydrophobic compounds thereby protecting cells from xenobiotics and other potential toxins. P-gp is expressed in numerous tissues including the small intestine, blood–brain barrier, hepatocytes, and kidney proximal tubule which affects intracellular and systemic drug concentrations; therefore, it is important to understand the behaviour of new drugs and therapies in regards to modification of P-gp.

P-gp belongs to the ATP-binding cassette (ABC) superfamily, which in humans, consists of several hundred transmembrane transport proteins which belong to 7 distinct subfamilies named ABCA – ABCG. They import and export a broad variety of substrates across cellular membranes including amino acids, lipids, drugs, and proteins. ABC genes are essential for many processes in the cell and mutations cause or contribute to several genetic disorders such as cystic fibrosis, neurological disease, retinal degeneration, Stargardt disease, cholesterol and bile transport defects, anemia, and abnormal drug responses). P-gp was the first identified human ABC transporter gene [25] and was first described in drug-resistant cells with a defined pattern of multidrug resistance [26]. P-gp relies on ATP as an energy source for drug efflux. Humans have two multidrug resistance (mdr) genes *mdr-1* and *mdr-2* (also called *mdr-3*), whereas rodents have three mdr genes *mdr-1A* (also called *mdr-3*), *mdr-1B* (also called *mdr-1*) and *mdr-2*. Only *mdr-1* in humans and *mdr-1A & B* in rodents are involved in drug transport and drug-resistance [27, 28]. Expression of P-gp in intestinal epithelial cells is responsible for efflux that limits cellular uptake into enterocytes and drug absorption. Drugs can be defined as P-gp inhibitors, P-gp enhancers or P-gp substrates. P-gp inhibitors impair P-gp mediated efflux while inducers enhance P-gp activity. A multitude of drugs, including several with a narrow therapeutic index, interact with P-gp and drug-drug interactions must be considered given their impact on bioavailability and therapeutic outcome. Hence, the FDA now advises drug developers to characterize interactions between new drugs and P-gp, as stated in guidelines released in 2012 concerning drug-drug interactions. This just signifies the importance for evaluating P-gp interactions during novel drug development and to recommend clinical guidelines for clinicians when administering P-gp substrate drugs.

We studied the *in vitro* interactions between two novel cholesterol lowering agents, DAPP and NSAS, and the P-gp transporter protein using Caco-2 cells.

## Materials and methods

### Materials

NSAS was provided by AMCOL International Corporation (Chicago, USA). Triton X-100, Tween-80, HEPES, Protease inhibitor cocktail, Na-deoxycholate, EDTA and NaCl were obtained from Sigma-Aldrich (St. Louis, MO, USA). All tissue culture reagents were from Invitrogen/Life Technologies (Grand Island, NY, USA). T-75 flasks, tissue culture treated plates and Transwell® inserts were from Corning Incorporated (Corning, NY, USA). CytoTox96® Non-Radioactive Cytotoxicity Assay, MTS CellTiter 96® AQ_ueous_ One Solution Cell Proliferation Assay and Pgp-Glo^TM^ Assay were from Promega Corporation (Madison, WI, USA). BCA^TM^ Protein Assay Kit was obtained from Pierce Biotechnology, Inc. (Rockford, IL, USA). NP-40 was purchased from Roche Applied Science and Trans-Blot® Transfer medium (nitrocellulose membrane 0.45 μM) from Bio-Rad (Hercules, CA, USA).

### Methods

#### Cell culture

Caco-2, human colon adenocarcinoma cells, were purchased from ATCC (Rockville, MD, USA) and passage numbers between 20 and 35 were used. Cells were cultured in Dulbecco’s modified Eagle’s medium (DMEM), supplemented with 10% fetal bovine solution (FBS), 292 μg mL^-1^ L-glutamine, 0.1 mM non-essential amino acids, 100 U/mL penicillin and 100 μg/mL streptomycin containing 1.5 g/L NaHCO_3_ at 37°C in humidified air containing 5% CO_2_. Stock cultures were grown in T-75 flasks. Once the cells reached a confluency of about 90%, they were then split by using 0.25% trypsin containing 1.0 mM EDTA. Cells were seeded in 96-well, 48-well, 12-well, or Transwell plates depending on the type of experiment. The medium was changed every other day.

#### Cytotoxicity measurements

To determine non-cytotoxic concentrations, the following markers of toxicity were employed: 1) cell plasma membrane integrity as determined by lactate dehydrogenase (LDH) release and 2) mitochondrial respiration as measured by the reduction of a tetrazolium compound (MTS) to a soluble formazan product. Caco-2 cells were seeded onto 96-well plates and were kept at 37°C in humidified air containing 5% CO_2_ until the cells reached 90% confluency. On the day of the experiment, the culture medium was exchanged for treatment solutions of 0 to 1000 μg/mL NSAS or 0 to 500 μM DAPP and 1% Triton X-100 (as positive control for cytotoxicity) in Hanks’ Balanced Salt Solution without phenol (HBSS) containing 10 mM HEPES, pH 7.4. LDH release (CytoTox96® Non-Radioactive Cytotoxicity Assay), MTS reduction (CellTiter 96® AQ_ueous_ One Solution Cell proliferation Assay) and bicinchoninic acid (BCA^TM^ Protein Assay Kit) assays were then performed, and cell viability was calculated relative to the 100% control (MTS-Assay). Cytoxicity was calculated relative to 100% cytotoxicity obtained from the Triton X-100 group (LDH-Assay).

#### Protein expression: western blotting

Protein levels were qualitatively observed by Western blot techniques. Caco-2 cells were treated with culture medium (control) or culture medium containing non-cytotoxic concentrations of NSAS or DAPP. Cells were washed three times with PBS and harvested with RIPA lysis buffer (50 mM HEPES, 150 mM NaCl, 2 mM EDTA, 0.5% Na-deoxycholate, 1% NP-40) containing protease inhibitor cocktail (1:100 dilution). Protein content was determined by BCA protein assay and the cell membrane proteins (20 μg/lane) were separated by electrophoresis through a 10% SDS-polyacrylamide gel and then electroblotted onto a nitrocellulose membrane. A pre-stained protein standard from Bio-Rad was used to identify the P-gp protein band at 170kD and actin at 42kD. The membrane was incubated overnight at 4°C in blocking buffer (1 × PBS, 2.5% nonfat dried milk, 0.1% Tween-20), and probed with a 1:300 dilution of primary antibody (C219 from Signet Pathology System Dedlam) to detect P-gp and a 1:1000 dilution of I-19 (Santa Cruz Biotechnology) to detect actin as an internal control. The membranes were washed 3 times with PBS and 1% Tween-20 (PBS-T) and the membrane was incubated in a 1:5000 dilution of anti-mouse IgG rabbit horseradish peroxidase (HRP)-conjugated antibody (Jackson ImmunoResearch Laboratories) and a 1:3000 dilution of anti-goat IRP bovine HRP (Santa Cruz Biotechnology) for P-gp and Actin respectively. Three washing steps with PBS-T followed. Bands were visualized with ECL and quantified with Labworks software (UVP).

#### RNA-isolation and RT-PCR

Caco-2 cells were harvested and total RNA was isolated with TRIzol® Reagent (Invitrogen) according to the manufactures instruction. RNA was reverse transcribed into cDNA. The concentration of cDNA reaction product was measured by using Oligreen-Assay (Molecular Probes). The primers were synthesized at the Oligonucleotide Synthesis Laboratory at UBC. Parameters and conditions for the tested primers were optimized. The following primers were used for the described studies. Mdr-1 (5′- GTC-ATT-GTG-GAG-AAA-GGA-AAT-CAT-G-3 and 5′- ATT-CCA-AGG-GCT-AGA-AAC-AAT-AGT-G-3′ and GAPDH (5′- TGA-AGG-TCG-GAG-TCA-ACG-GAT-3′ and 5′- TCG-CTC-CTG-GAA-GAT-GGT-GAT-3′). A sample from each PCR product was subjected to electrophoresis on a 1.5% agarose gel (containing Ethidium bromide). A 100 bp ladder was used to identify the size of PCR products. The fluorescent bands were imaged under UV light (UV-Epi Chemi II) and quantified with UVP-labworks software.

#### Transmembrane transport of rhodamine 123

Caco-2 cells form monolayers and expressexpress P-gp on their apical membranes thereby differentiating into a highly functionalized epithelial barrier that is morphologically and biochemically similar to small intestine columnar epithelium. A good system to measure apical and basolateral transport across monolayers and to determine the permeability of a certain substance is a Transwell® plate. Cells are grown on a semi-permeable membrane which is placed between two chambers. Donor and acceptor chambers are determined by experiment type. To model intestinal drug absorption, the substance is given into the apical side and the concentration in the basolateral side is measured. Secretory transport can be determined if the substance is added onto the basolateral side and the concentration in the apical chamber is measured. P-gp mediated transport is characterized by basolateral to apical transport greater than apical to basolateral transport.

Caco-2 cells were seeded in polycarbonate membrane Transwell® plates. at a densitiy of 40.000 cells/cm^2^ and grown in a humidified chamber (at 37°C, of 5% CO_2_.) with media changes every 2 days. The growth media (Dulbecco’s minimal essential medium-DMEM) contained 10% heat-activated fetal bovine serum, 292 μg/ml glutamine, 0.1 mM non-essential amino acids, 100U/ml penicillin and 100 mg/ml glutamine. For the treatment experiments, NSAS or DAPP in different concentrations was added to the media and either applied onto apical and/or the basolateral side of the Transwell plate.

Transepithelial electrical resistance (TEER) of the monolayers was measured to confirm monolayer integrity with a Millicell Electrical Resistance System (Milipore Corp., Bedford, MA). Caco-2 cells with TEER Values above 300 Ω · cm^2^ were used for transport studies. Cells were washed 3 times with PBS before treatment solutions were loaded on the apical side. Plates were incubated and TEER values were measured before and after the treatment to ensure integrity of the monolayer and tight junctions.

Rhodamine 123 (Rh123) is a fluorescent dye and P-gp substrate and has been used as a probe to measure the functional activity of P-gp. It has a molar extinction coefficient of 85,200 M^-1^ cm^-1^ at 511 nm. Rh123 was added to either the apical or basolateral chamber, with transport buffer in the corresponding receiver chamber. In a time dependent manner, samples (50 μl) were collected with media replacement from receiver chamber and transferred into a 96-well plate. Rh123 concentrations were measured with a Fluoroskan Ascent fluorometer (excitation = 485 nm and emission = 538 nm).

The apparent permeability P_app_ can be determined by using the following equation:


A is the area of absorption (cell monolayer), dQ/dt is the cumulative amount of test compound appearing in the receiver compartment of the assay system versus time, and C_o_ is the initial concentration of the test compound in the donor compartment.

#### Statistical analysis

All data sets were analyzed for statistical significance by parametric methods using SigmaStat version 3.5. When comparisons were made between two groups, unpaired two-tailed *t* tests were used. All data are expressed as mean ± standard deviation.

## Results

### Measurement of toxicity of NSAS and DAPP in Caco-2 cells

**Figure 3:**
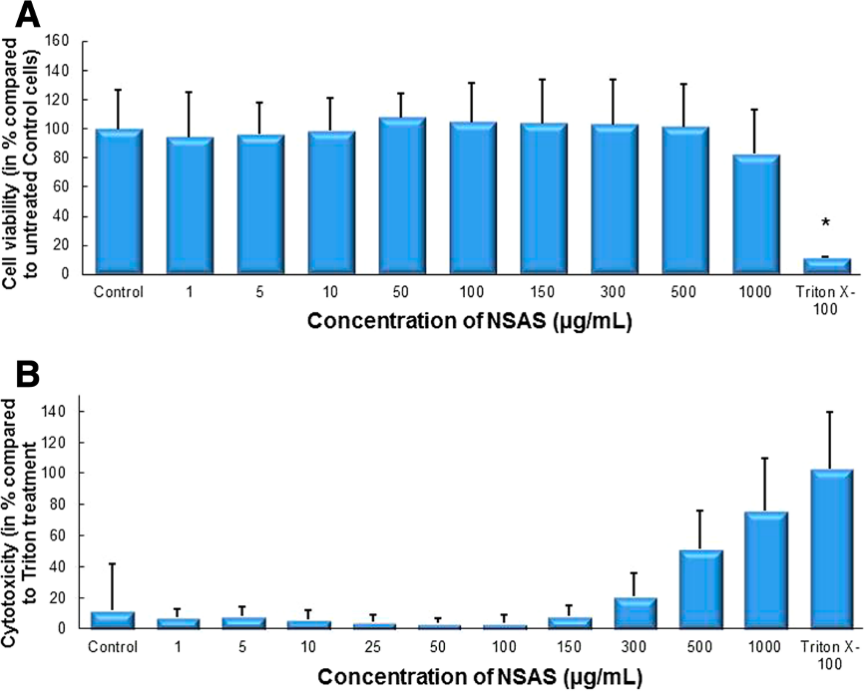
**Cell viability and cytotoxicity after treatment with NSAS.** Effect on Caco-2 cells. MTS-Assay **(A)** and LDH-Assay **(B)** after incubation with different concentrations of NSAS for 24 hours. Results are compared to untreated control cells (in A) and Triton X-100 (in B). Each bar represents the mean ± SD. *p < 0.05. N = 3.

**Figure 4:**
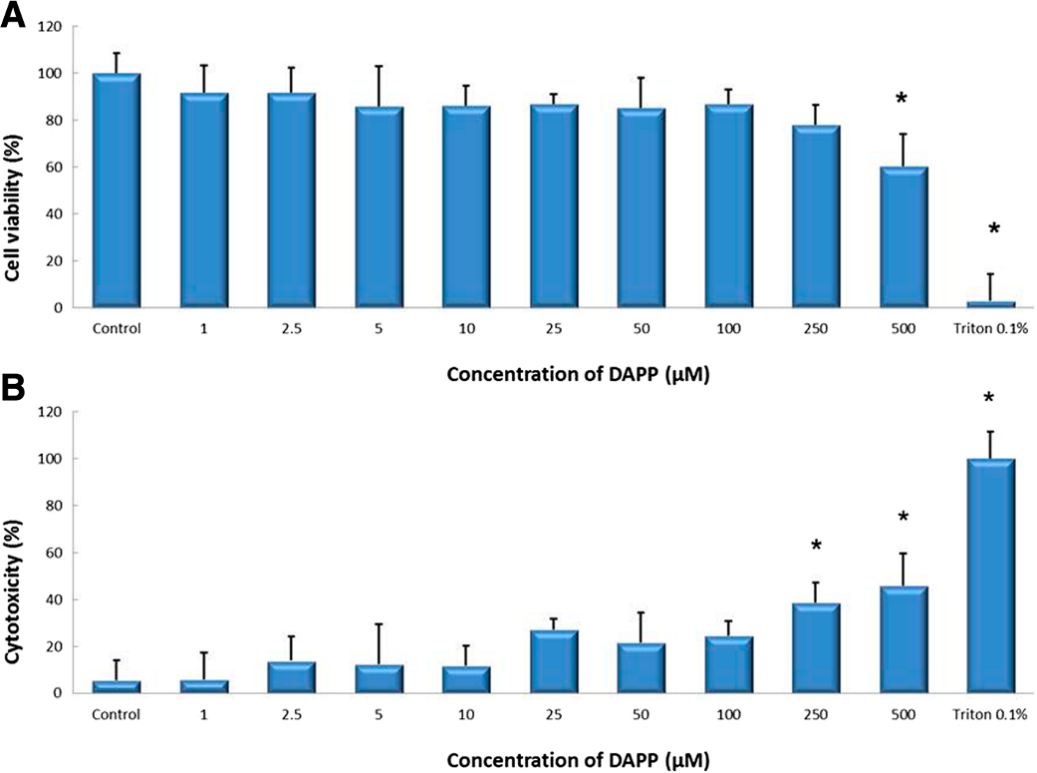
**Cell viability and cytotoxicity after treatment with DAPP.** Effect on Caco-2 cells. MTS-Assay **(A)** and LDH-Assay **(B)** after incubation with different concentrations of DAPP for 24 hours. Results are compared to untreated control cells (in A) and Triton X-100 (in B). Each bar represents the mean ± SD. *p < 0.05. N = 4.

Treatment with concentrations between 0 and 500 μg/ml NSAS and 0 and 250 μM DAPP showed no significant difference in cell viability compared to untreated control as measured by MTS assay (Figures 3A and 4A). Results from the LDH assay showed no significant cytoxicity for concentrations between 0 and 300 μg/mL NSAS and 0 and 100 μM DAPP compared to untreated control (Figures 3B and 4B). These nontoxic concentrations were subsequently used to treat cells for cholesterol uptake, transporter protein expression, Rh123 accumulation and transport studies across the Caco-2 cell monolayer.

### P-gp protein expression

**Figure 5:**
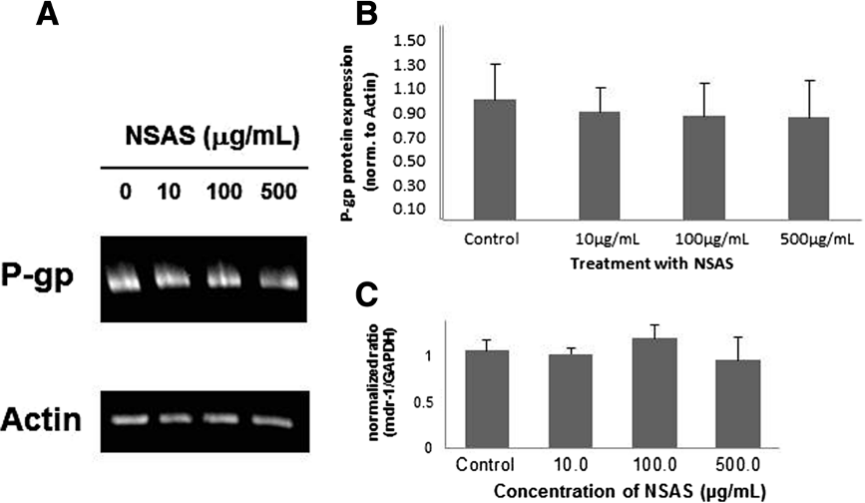
**P-gp protein expression in Caco-2 cells after treatment with NSAS.** Cells were exposed for 24 hours to media alone (control), 10 μg/mL, 100 μg/mL and 500 μg/mL NSAS compound A. **(A)** Representative Western Blot. **(B)** The data represent the mean ± standard deviation of P-gp protein normalized by the protein expression of actin. There is no statistically significant difference between treatment groups and control. N = 3. **(C)** Expression profile of *mdr-1* gene expression in Caco-2 cells. A sample from each PCR product was subjected to electrophoresis on a 1.5% agarose gel and the fluorescent bands were quantified with UVP-Labworks software. Each value represents the mean ± standard deviation of N = 3.

**Figure 6:**
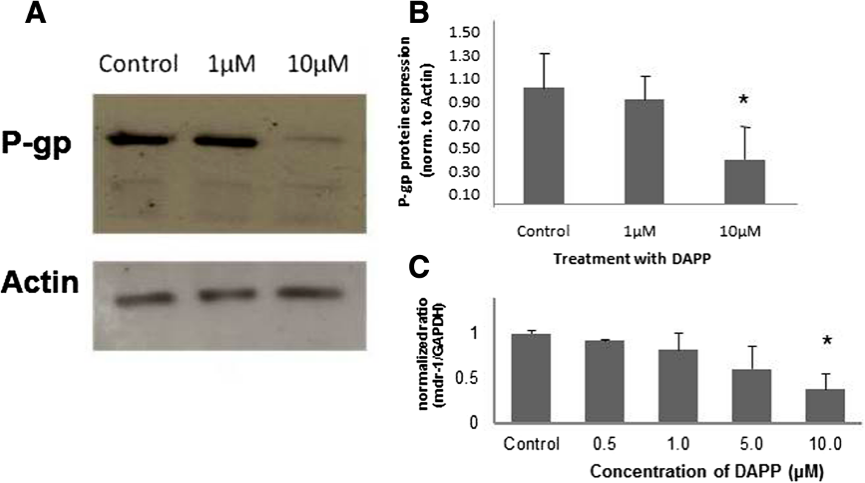
**P-gp protein expression in Caco-2 cells after treatment with DAPP.** Cells were exposed for 24 hours to media alone (control), 1 μM, 10 μM and 500 μM DAPP. **(A)** Representative Western Blot. **(B)** The data represent the mean ± standard deviation of P-gp protein normalized by the protein expression of actin. *p < 0.05. N = 6. **(C)** Expression profile of *mdr-1* gene expression in Caco-2 cells. A sample from each PCR product was subjected to electrophoresis on a 1.5% agarose gel and the fluorescent bands were quantified with UVP-Labworks software. Each value represents the mean ± standard deviation of N = 6. *p < 0.05.

No changes in protein expression for P-glycoprotein were measured when Caco-2 cells were incubated for 24 hours with NSAS at 10 μg/mL, 100 μg/mL, and 500 μg/mL (Figure 5). However when Caco-2 cells were treated for the same length of time with DAPP a significant change was observed (Figure 6). Incubation with DAPP 10 μM leads to a lesser expression of P-gp (41 ± 6% versus untreated control cells). We performed PCR reactions for those tested time points. A significant decrease in *mdr-1* gene expression was observed when cells were treated with DAPP. All data obtained for mdr-1 gene expression were normalized for GAPDH gene concentration (Figures 5C and 6C).

In summary, down-regulation of *mdr-*1 gene expression was shown, complementing the results for decreased P-gp protein expression (38.5 ± 17% for treatment with 5 μM DAPP and 61.2 ± 25% for treatment with 10 μM DAPP versus untreated control cells). Figure 6 shows the correlation from a decreased gene expression into decreased protein expression even after one week of treatment. The largest effect was seen for a concentration of 10 μM of DAPP.

### Transmembrane transport of rhodamine 123

Experiments were performed to measure the activity of P-gp in Caco-2 cells after treatment with DAPP.

**Figure 7:**
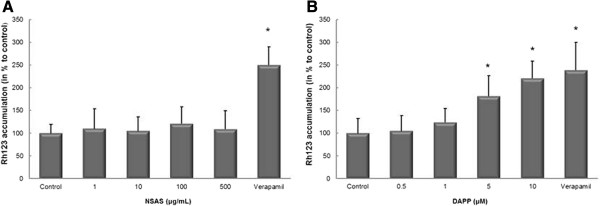
**Effect of NSAS and DAPP on Rhodamine 123 uptake in Caco-2 cells.** Cells were exposed for 24 h to media alone (control), or to increasing concentrations of NSAS **(A)** or DAPP **(B)**. Cellular accumulation of Rhodamine 123 was normalized with respect to the protein content in each well. Data is presented as mean ± standard deviation with N = 6. *p < 0.001 vs. control **(A)** and *p < 0.05 vs. control **(B)**.

We observed a significant down-regulation of *mdr-1* m-RNA (e.g. 38.5 ± 17% for treatment with 5 μM DAPP and 61.2 ± 25% for treatment with 10 μM DAPP; n = 6, P* < 0.05) within Caco-2 cells. Western Blot analysis of P-glycoprotein expression showed that changes in *mdr-1* gene expression lead to correlating changes in P-gp protein expression. This down-regulation of P-glycoprotein also resulted in a decreased activity of P-glycoprotein compared to untreated control.Treatment with 5 and 10 μM DAPP resulted in a significant increase in Rh123 accumulation by two-fold relative to untreated control cells (Figure 7B). Accumulation of Rh123 in those Caco-2 cells is similar to Rh123 accumulation in cells treated with the positive control for P-gp inhibition, verapamil.

**Figure 8:**
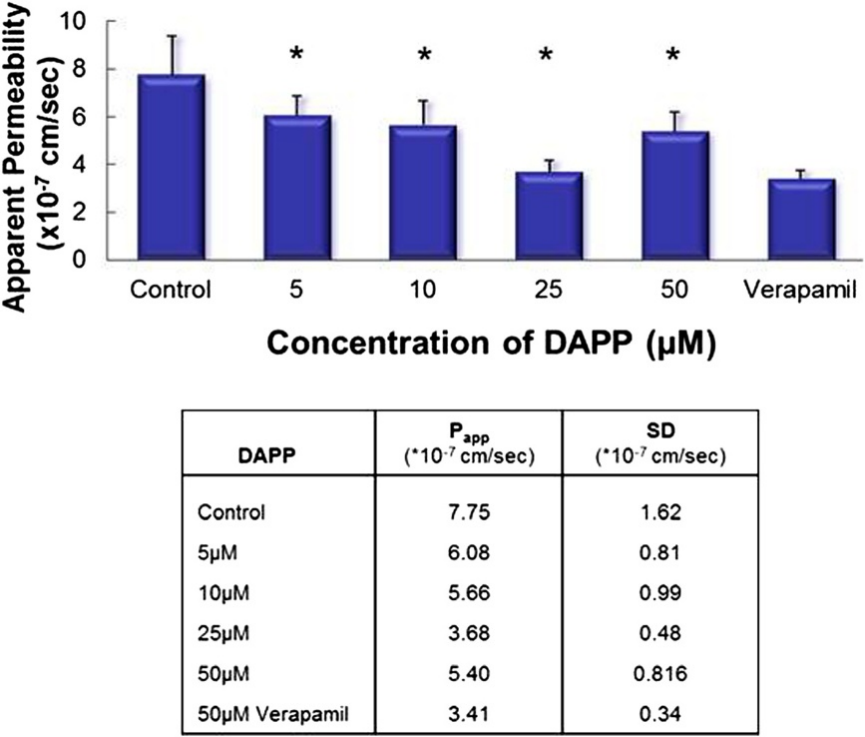
**Effect of pre-incubation with DAPP on P-gp transport of Rhodamine 123 across Caco-2 cell monolayer (basolateral to apical transport) and apparent Permeability Papp.** Data represent the average of N = 6 ± SD. *p < 0.025. TEER values were measured before and after the treatment and at the end of the experiment. No changes in TEER values were seen which indicates the integrity of the monolayers. Treatment with DAPP lead to a statistically significant decrease in secretory flux compared with untreated control cells. Treatment with Verapamil represents a positive control for P-gp inhibition.

These findings suggest that DAPP not only has cholesterol-lowering properties but also decreases P-pg mediated drug efflux and might reverse multi-drug resistance.DAPP treatment lead to a statistically significant decrease in secretory flux compared with untreated control cells (Figure 8), while TEER values stayed unchanged.Bentonite on the other hand had shown cholesterol lowering characteristics without influencing transporter proteins. Also, no changes for Rh123 accumulation were observed (Figure 7A) when compared to untreated control cells.

In Summary DAPP has an influence on the expression of *mdr-1.;* a decrease of the mRNA level lead to a decrease in P-glycoprotein expression. A corresponding decreased P-gp activity was also observed. None of those changes in *mdr-1* gene expression, P-gp protein expression and activity were seen with NSAS.

## Conclusions

Since the introduction of statins in the 1980s, they have become a golden standard of lipid-lowering therapy. They are prescribed for treatment of hypocholesterolemia and to reduce LDL-cholesterol and therefore reducing coronary artery disease-related morbidity and mortality. Over the past years statins have been proved to be safe and well-tolerated drugs. However there are more and more reports on patients who either have an intolerance to statin treatment or experience minor to severe adverse reactions. The need for new and alternative therapies is more present and prominent. Studies of either new pharmacological agents or treatments in combination with established therapies are under way.

The study here investigates the effect on P-gp expression and activity of two substances, DAPP and NSAS, of the new class of cholesterol lowering agents.

This is of relevance for drug toxicity and efficacy since most patients take several medications simultaneously and many of the commonly prescribed drugs are substrates for P-gp. Drugs with narrow therapeutic indexes have shown large increases in concentration when co-administred with potent P-gp inhibitors or when the drug itself functions as P-gp inhibitor.

In previously published studies both agents (DAPP and NSAS) have shown a significant decrease in cholesterol absorption. This was tested in different models and no severe side effects were observed.

We found that unlike DAPP, NSAS did not change P-gp expression in our Caco-2 cell model. From that perspective NSAS may be a safer candidate for cholesterol-lowering treatment with regards to drug-drug interactions and adverse effects. Given our observation, DAPP has the potential to modulate P-gp mediated drug transport. Phytostanols and phytosterols have been widely used as additives to cereals, margarine, drinks and other food sources or are given as supplements to reduce serum cholesterol levels. Given the new findings of lowering P-gp protein expression and activity, this observation raises the possibility that when given with other drugs, they might impact uptake, bioavailability, and therapeutic outcomes.

There are several limitations to this set of studies. Further studies are required to determine the interactions between these two agents and other ABC transporter proteins. Though P-gp is the most significant one, there are several other transporter proteins which may impact drug absorption such as multidrug resistance associated protein (MRP), breast cancer resistance protein (BCRP), organic anion transporter (OAT), organic anion transporting polypeptide (OATP), organic cation transporter (OCT) and oligopeptide transporter (PEPT). Additionally, these finding need to be confirmed in an animal model to evaluate clinical significance of our findings.

Data presented here further substantiate the need for addressing and investigating potential transporter protein interactions when developing new drug therapies.
